# Intercropping Competition between Apple Trees and Crops in Agroforestry Systems on the Loess Plateau of China

**DOI:** 10.1371/journal.pone.0070739

**Published:** 2013-07-25

**Authors:** Lubo Gao, Huasen Xu, Huaxing Bi, Weimin Xi, Biao Bao, Xiaoyan Wang, Chao Bi, Yifang Chang

**Affiliations:** 1 College of Water and Soil Conservation, Beijing Forestry University, Beijing, P.R. China; 2 Key Laboratory of Soil and Water Conservation, Ministry of Education, Beijing, P.R. China; 3 Department of Biological and Health Sciences, Texas A&M University-Kingsville, Kingsville, Texas, United States of America; United States Department of Agriculture, United States of America

## Abstract

Agroforestry has been widely practiced in the Loess Plateau region of China because of its prominent effects in reducing soil and water losses, improving land-use efficiency and increasing economic returns. However, the agroforestry practices may lead to competition between crops and trees for underground soil moisture and nutrients, and the trees on the canopy layer may also lead to shortage of light for crops. In order to minimize interspecific competition and maximize the benefits of tree-based intercropping systems, we studied photosynthesis, growth and yield of soybean (*Glycine max* L. Merr.) and peanut (*Arachis hypogaea* L.) by measuring photosynthetically active radiation, net photosynthetic rate, soil moisture and soil nutrients in a plantation of apple (*Malus pumila* M.) at a spacing of 4 m × 5 m on the Loess Plateau of China. The results showed that for both intercropping systems in the study region, soil moisture was the primary factor affecting the crop yields followed by light. Deficiency of the soil nutrients also had a significant impact on crop yields. Compared with soybean, peanut was more suitable for intercropping with apple trees to obtain economic benefits in the region. We concluded that apple-soybean and apple-peanut intercropping systems can be practical and beneficial in the region. However, the distance between crops and tree rows should be adjusted to minimize interspecies competition. Agronomic measures such as regular canopy pruning, root barriers, additional irrigation and fertilization also should be applied in the intercropping systems.

## Introduction

The Loess Plateau is the birthplace of China's primitive agriculture. However, because of unsound land use and destruction of forests, the Loess Plateau has suffered serious soil erosion. At the same time, rapid population growth has also brought greater pressure to the environment in the region. The ensuing ecological and environmental problems have slowed down the economic development and living standards of local people. These problems lead to further deterioration of ecological environment, forming a vicious cycle. The local government is facing dual pressures from both economy and ecology.

Agroforestry systems have been considered as an effective practice to alleviate the conflicts between the rapidly growing population and the limited arable land resources [1], [2]. In recent years, agroforestry management has been widely applied in the Loess Plateau region for reducing soil erosion and water loss, restoring ecological balance, raising land utilization rate and increasing economic benefits [3], [4]. However, in most agroforestry systems, competition for light, moisture and nutrients exists at the interface between trees and crops which can cause a reduction of crop yield [5]. It is a major constraint that has affected stability of the structure and the function of the agricultural ecosystems. The competition between woody tree species and understory crop species not only exists aboveground (competition for light) but also comes from belowground (competition for soil moisture and nutrients), leading to lower crop yield. According to Friday and Fownes, the competition between trees and crops is overwhelmingly for light which is the main reason for the reduction of maize in alley cropping system in Hawii, USA [6]. Similar results were reported by Peng et al. in loess area of Weibei in Shaanxi Province, China [7]. Elsewhere in southern Australia, studies showed that reduced crop yields are associated with the competition for water in windbreak and alley systems [8], [9]. Kowalchuk and Jong found that, especially in drought years, competition for water is the principal factor affecting the yield of spring wheat intercropped with shelterbelts in Western Saskatchewan [10]. In some related studies, the results indicated that competition for nutrients does not exist in intercropping systems [11]–[13]. However, others reported that as one of the main reasons leading to the reduction of crop yield, the competition for soil nutrients does exist in the interface of trees and crops and has a negative impact [14], [15]. It is very important to explore the competitive mechanism in intercropping systems, in order to provide optimum management strategies and technologies for managing intercropping system with high-yield, high-efficiency and stabilization.

Apple-crop intercropping system is one of the most commonly applied agroforestry systems in the Loess Plateau region owing to its good ecological, social and economic benefits. However, only few studies focused on this intercropping system in the area. In order to explore the biological reasons of the competition in typical intercropping systems and to provide effective management techniques, we report on a study of two apple-crop intercropping systems (apple-soybean, apple-peanut) on the Loess Plateau region in the western portion of Shanxi Province. The objectives of our research were (1) to analyze the interspecies competition relationship between trees and crops; (2) to find the limiting factors in the development of intercropping systems in this area; (3) to offer possible solutions to minimize the interspecies competitions and maximize resource utilization; (4) to enrich the related study and to improve the management of the intercropping systems in this region.

## Materials and Methods

### Study site

The study site was located in the Baidong Village, Jixian County, Shanxi Province, China (36°06′ N, 110°35′ E, 1025 m a.s.l.). The area is a typical hill and gully region of the Loess Plateau. The annual mean rainfall is about 575 mm, and the mean annual temperature is 10°C (1991–2010). The precipitation is unevenly distributed seasonally, with an average rainfall of 463 mm from June to August (1991–2010), which contributed about 80% of annual precipitation. The parent material of the soil is loess, and the soil properties are uniform. The bulk density, pH, total porosity, CaCO_3_ content, cation exchange capacity, organic C, total N and available P of the top soil layer (100 cm) were 1.32 Mg•m^−3^, 8.24, 50.16%, 18.35%, 18.43 cmol•kg^−1^, 6.27 g•kg^−1^, 0.39 g•kg^−1^ and 4.39 mg•kg^−1^, respectively. The main intercropping tree species are Apple (*Malus pumila* M.), Apricot (*Prunus armeniaca* L.), Pear (*Pyrus bretschneideri* R.), Chinese arborvitae (*Platycladus orientalis* (L.) and Franco) and Black locust (*Robinia pseudoacacia* L.).

### Ethics Statement

No specific permits were required for the described field studies. The sampling locations were not privately-owned or protected in any way and the field studies did not involve endangered or protected species.

### Treatments and Crop Cultivation

Two typical intercropping systems of apple-soybean and apple-peanut were chosen for this study during the crop growing season of 2011 and 2012. The apple trees were planted in an East-West orientation in 2007. The characteristics of the apple trees intercropped with soybeans and peanuts in July 2011 are listed in Table 1. There were four treatments in this study: apple-soybean intercropping treatment (AS), soybean monoculture served as control (CS), apple-peanut intercropping treatment (AP) and peanut monoculture served as control (CP). Each treatment had three replicates. Each replicate of intercropping treatment (AS and AP) was an 8 × 10 m plot that included 12 trees planted in three rows with 4 m between trees and 5 m between rows. Each replicate of control treatment (CS and CP) was the same size of 8 × 10 m. For all treatments, the crops were planted at a spacing of 0.4 m with in rows and 0.5 m between rows and received the same agricultural management practices. Soybean and peanut were grown 0.3 m from an adjacent tree row in the intercropping systems. All plots received 147 kg N ha^−1^, 30 kg P ha^−1^ and 30 kg K ha^−1^ as basal fertilizer and no additional fertilizer or irrigation in the rest of the year.

**Table 1:** Characteristics of apple trees intercropped with soybean and peanut in the experimental sites in July 2011.

Measurement	Intercropped with soybean	Intercropped with peanut
Tree height (m)	2.4	2.5
DBH (cm)	4.1	4.2
Depth of live crown (m)	1.7	1.8
Mean radius of crown (m)	1.3	1.2

### Measurements of Plant Photosynthesis, Soil Moisture and Nutrients

**Figure 1:**
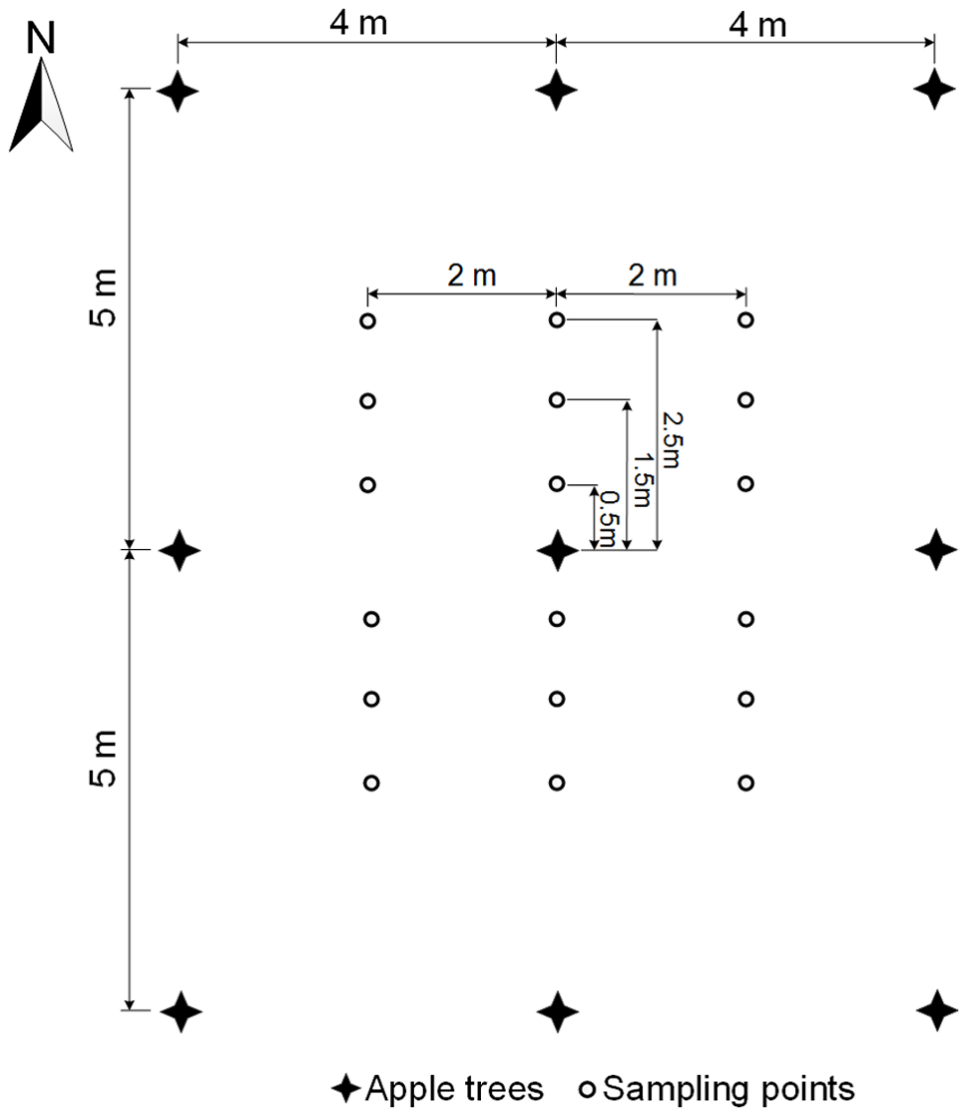
Sampling points of plant photosynthesis, soil moisture and nutrients in the intercropping study sites.

For the sampling of plant photosynthesis, soil moisture and soil nutrients, six sampling locations at distances of 0.5 m, 1.5 m and 2.5 m, respectively, from both side of tree row were identified as sampling points in each intercropping plot (Figure 1). The sampling points were further divided into three equal groups and denoted as F0.5, F1.5 and F2.5 based on the distance (0.5 m, 1.5 m and 2.5 m) from the tree row. Measurement parameters of F0.5, F1.5 and F2.5 were used to represent the major locations of 0.5 m, 1.5 m and 2.5 m away from apple tree row. For each control plot, five selected points were established with an S-shaped sampling method.

Photosynthetically active radiation (PAR) and net photosynthetic rate (NPR) of crops were performed by two portable Li-6400 photosynthesis systems which had a 6 cm^2^ clamp-on leaf chamber connected to the main engine (Li-6400, Li-Cor Inc., Lincoln, NE, USA) under ambient humidity, temperature and irradiance. One fully expanded leaf from the upper part of the crop canopy in each sampling point was selected and measured five times with 2 h intervals during daytime (0900–1700 h). During each measurement period, all sampling points of intercropping treatment and control treatment were visited. These treatments were measured in mid-August 2011 and again in late August 2012, the typical phenological phases of peanut and soybean. For all measurements, the flow velocity was set at 500 µmol•s^−1^ and the airstream entering the chambers was kept at the growth CO_2_ concentration (370 µmol•mol^−1^) by a computer-controlled CO_2_ injector system supplied with Li-6400. PAR and CO_2_/H_2_O exchanged by the leaf were measured concurrently with the quantum sensor and the infrared gas analyzer on LI-6400. The data were recorded and calculated automatically with the software in the photosynthesis system.

For soil moisture, the samples were taken at different phenological phases of soybean and peanut: 8 July, 23 August, and 23 September in 2011; and 4 July, 11 August, and 22 September in 2012. A drill was used to remove the soil from 0–100 cm in 20 cm intervals in soil profile. The soil moisture content was determined gravimetrically in each layer. The mean soil moisture content of the five layers in all sampling time (2011 and 2012) was calculated and used as the final value of the sampling point.

For the sampling of soil nutrients, the soil samples were taken on 23 August, 2011 and 11 August, 2012, the typical phenological phases of peanut and soybean. The soil samples were collected from a depth of 0–100 cm in soil profile with a drill. Organic matter content was determined by H_2_SO_4_-K_2_Cr_2_O_7_ pyrogenation. Total N was determined using the Kjeldahl method, with a KDY-9830 N Analyzer. Available P was determined by Olsen sodium-bicarbonate extraction. Available K was determined by flame photometer.

### Measurements of Crop Growth and Yields

For the sampling of crop growth, we used the same sample locations as for soil moisture. A single crop plant was sampled at each sampling point on 24 August 2011 and 12 August 2012. A total of 69 soybean plants and 69 peanut plants were harvested during each measurement period. In the lab, plant height, hundred leaf dry weight and total above-ground biomass of all plants were measured and recorded.

At the end of the growing season, in each intercropping plot, peanuts and soybeans were harvested from both sides of the tree row in two rectangular areas. The rectangular area was 4.0 m long and 2.7 m wide. As the convenience of the study, the two rectangular areas were divided into three groups: (1) the area of 0.3–1.0 m away from tree row; (2) the area of 1.0–2.0 m away from tree row; and (3) the area of 2.0–3.0 m away from tree row. The yields of the three groups were used to represent the crop yield of F0.5, F1.5 and F2.5, respectively. In the control plots, 2 m × 2 m quadrates of soybean and peanut were harvested to get the grain production. The peanuts and soybeans were dried at 70 °C and then weighed to obtain an average dry weight. Yield values were reported on a per hectare basis.

### Data analysis

All parameters (PAR, NPR, soil moisture, soil nutrients content, crop growth and yields) measured for control treatments and three major locations (F0.5, F1.5 and F2.5) of intercropping treatments were described in terms of mean values followed by respective standard deviations. Simple regression analysis was used to examine the relationships between the data of PAR, NPR, soil moisture and the distance from the tree row. Differences among groups for each crop (soybean or peanut) were determined by one-way ANOVA, and the results of the multiple comparisons were performed with least significant difference (LSD) test at *P*<0.05. NPR, total above-ground biomass and yield values of soybean and peanut had a correlated analysis with environmental parameters to decide the effect of apple trees competition on crop growth and productivity via bivariate correlation (Pearson) analysis at *P*<0.05 and *P*<0.01. All the analyses were performed by using the software IBM SPSS Statistics 20.0 for Windows.

## Results

### Light Interception and Plant Photosynthesis

**Figure 2:**
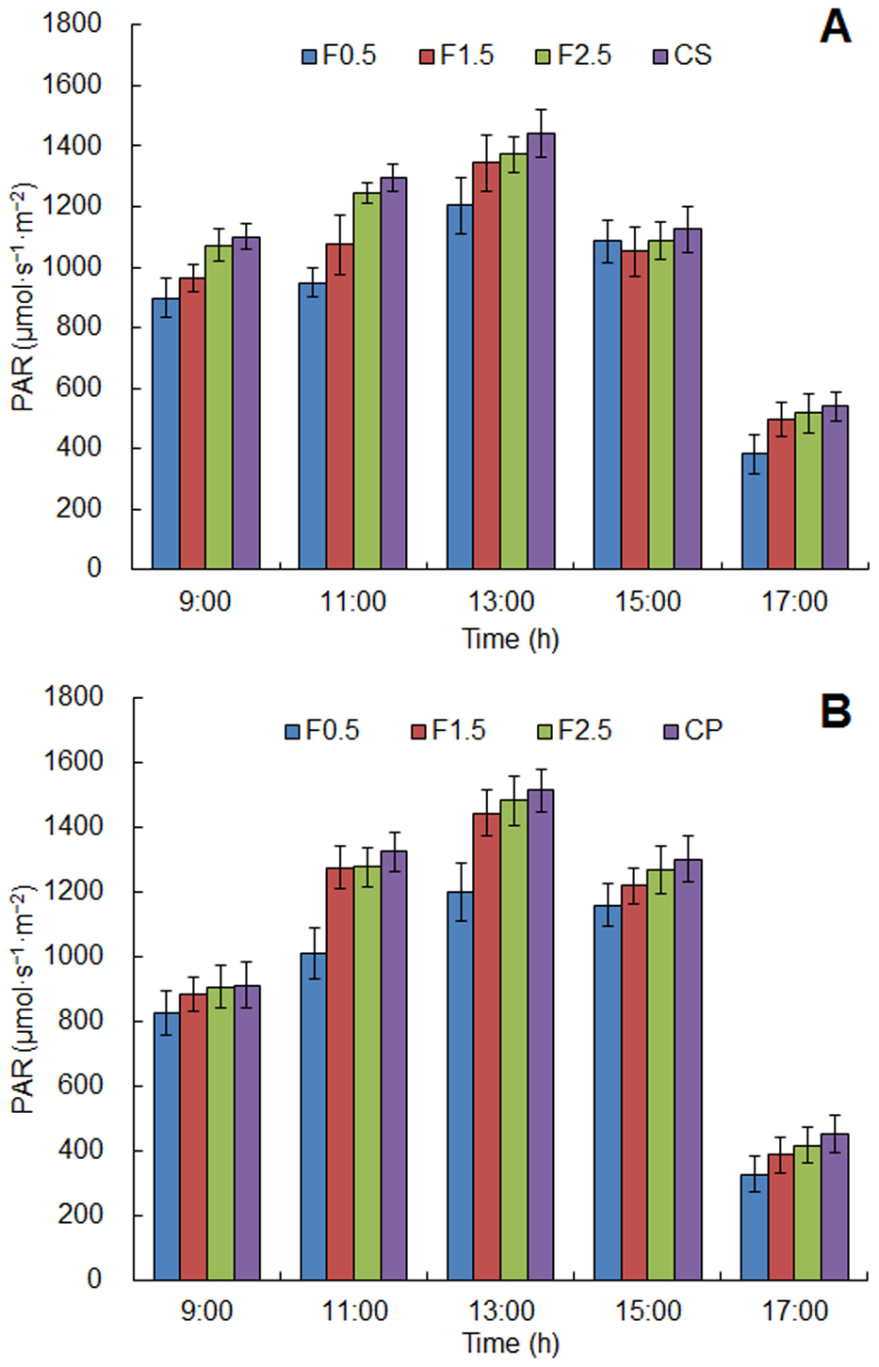
Diurnal variation of photosynthetically active radiation (PAR) for the intercropping systems and its control (A. apple–soybean and B. apple–peanut). F0.5, F1.5 and F2.5 were used to represent the sampling points which had different distance (0.5 m, 1.5 m and 2.5 m) from the tree row. Error bars indicate standard deviation.

**Figure 3:**
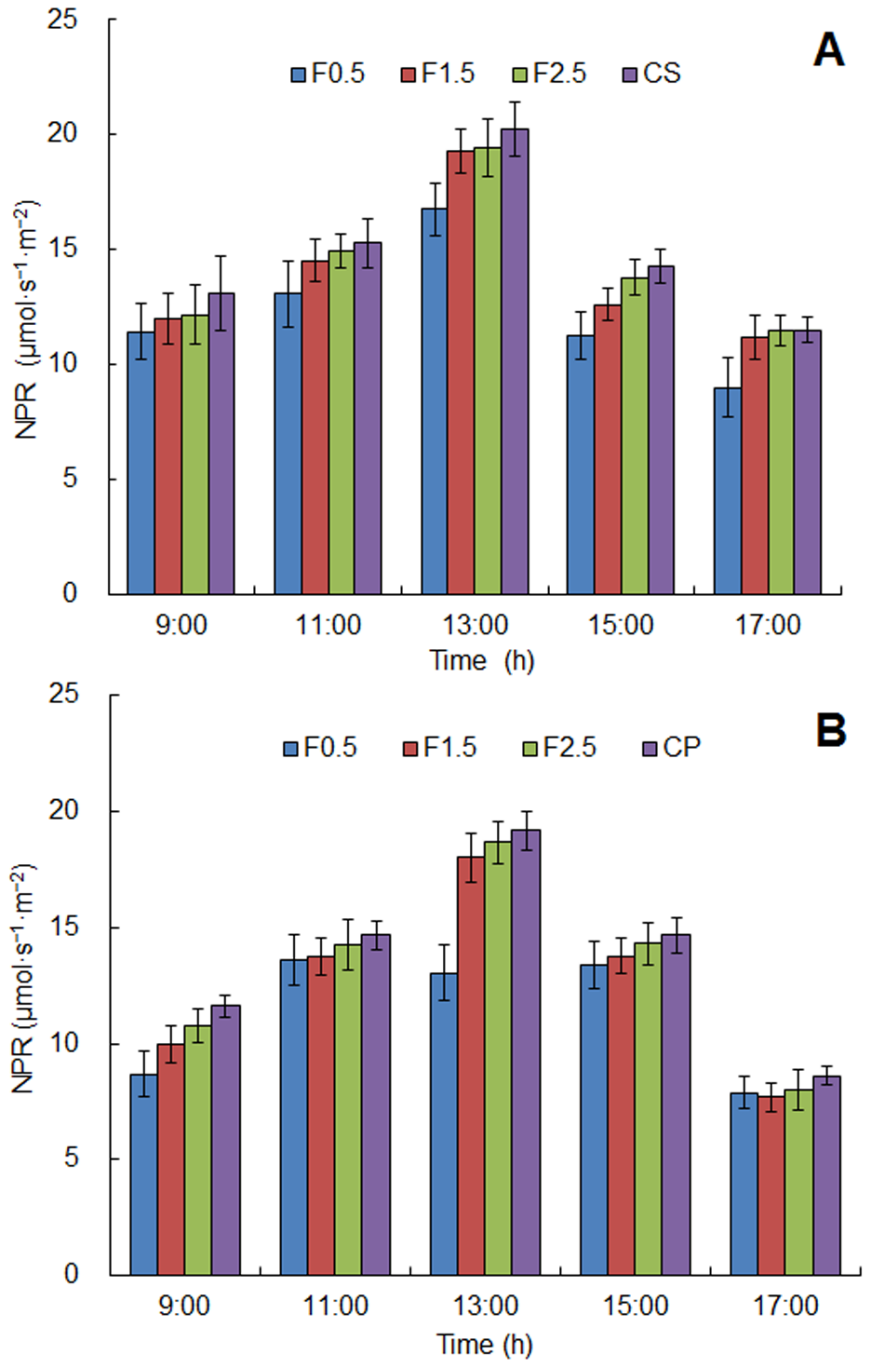
Diurnal variation of net photosynthetic rate (NPR) for the intercropping systems and its control (A. apple–soybean and B. apple–peanut). F0.5, F1.5 and F2.5 were used to represent the sampling points which had different distance (0.5 m, 1.5 m and 2.5 m) from the tree row. Error bars indicate standard deviation.

For both crops, diurnal variation of photosynthetically active radiation (PAR) in the intercropping systems and the monoculture configurations (control treatments) showed a single peak curve with time (Figure 2). The peak of PAR appeared at 13:00 pm and the minimum value appeared in 17:00 pm. Because of reflectance, absorbance and transmittance by the apple tree canopy, the PAR of crops in the intercropping systems were lower than that in the monoculture configurations during the same period. On the horizontal distribution, the general trend was that the closer the crops to the tree rows, the lower the PAR received. The same tendency was found in diurnal variation of net photosynthetic rate (Figure 3).

**Figure 4:**
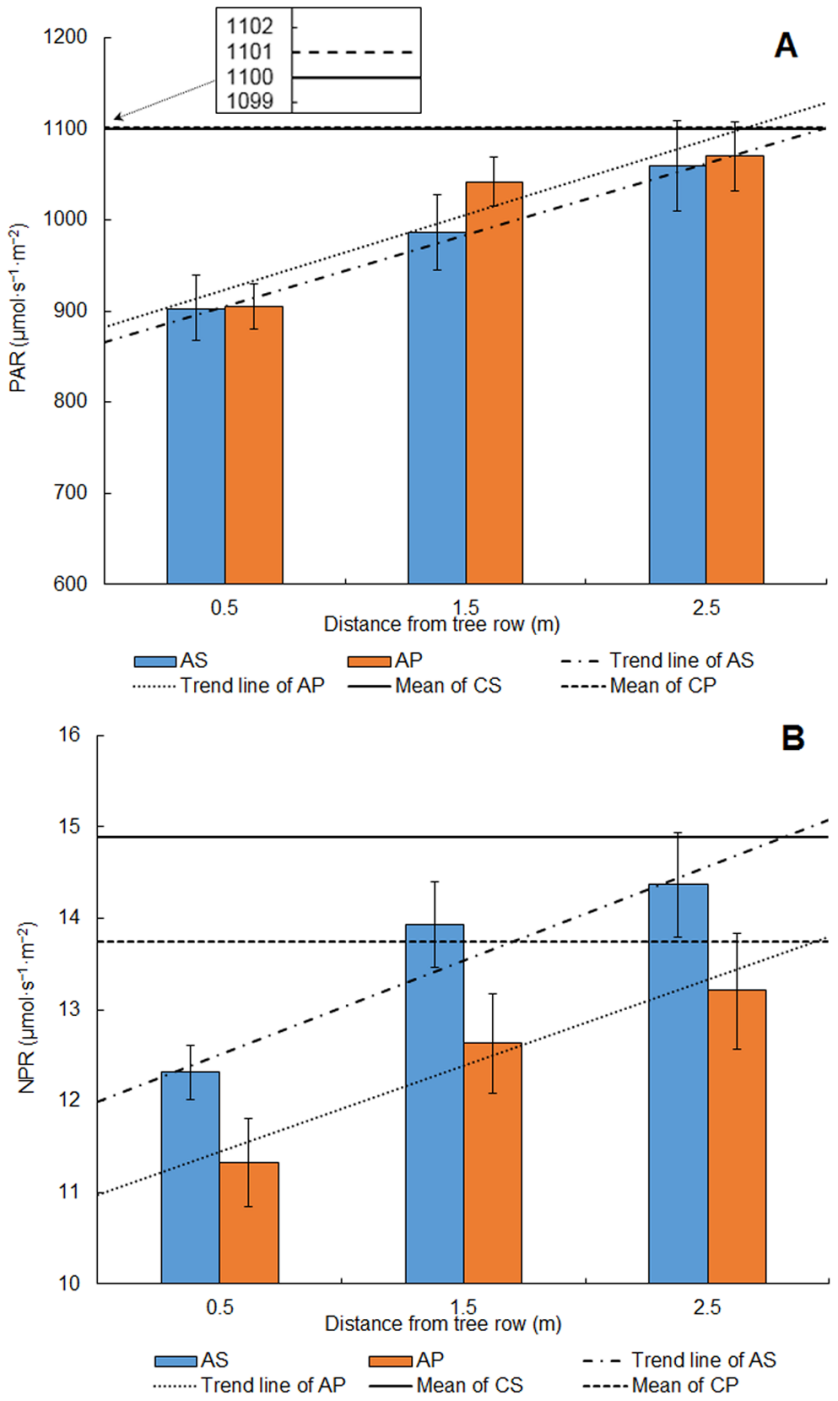
Daily mean of photosynthetically active radiation (PAR) and net photosynthetic rate (NPR) for the intercropping systems and its control (A. PAR and B. NPR). Vertical lines indicate confidence interval at 95% level.

The daily mean values of PAR showed a clear linear relationship with distance from the apple tree row in both intercropping treatments (Figure 4A). The trend lines of PAR (*Y*, µmol•s^−1^•m^−2^) and distance from trees rows (*X*, m) were *Y* = 78.5×+865.3 (*R* ^2^ = 0.999) in apple-soybean intercropping treatment (AS) and *Y* = 82.5×+881.9 (*R* ^2^ = 0.873) in apple-peanut intercropping treatment (AP). The slopes of both regression lines suggested that the PAR in AP treatment had a higher growth than that in AS treatment as the distance from the tree increased. As shown in Figure 4A, PAR reaching the upper parts of the crop canopy in AP treatment also had higher values than that in AS treatment at the same distance away from the tree row. It indicated that peanut canopy could obtain more solar radiation in AP treatment than soybean canopy in AS treatment. At confidence level of 95%, the control treatment PAR mean fell within the confidence intervals of F2.5 in the corresponding intercropping systems. Compared with the corresponding control treatment, PAR at F0.5 and F1.5 showed a reduction of 17.9% and 10.4% in AS treatment, respectively, 17.8% and 5.4% in AP treatment. Similar linear relationships were also obtained through regression analysis of the relationship between NPR and distance from the apple tree row (Figure 4B). The trend lines of NPR (*Y*, µmol•s^−1^•m^−2^) and distance from trees rows (*X*, m) were *Y* = 1.025×+12.003 (*R* ^2^ = 0.902) in AS treatment, and *Y* = 0.940×+10.983 (*R* ^2^ = 0.951) in AP treatment. The NPR in AS treatment had higher values and growth than that in AP treatment as the distance from the tree increased which was different from the measurement of PAR. The control treatments mean fell within the confidence intervals of F2.5 in the corresponding intercropping systems at confidence level of 95%.

### Spatial Distribution of Soil Moisture

**Figure 5:**
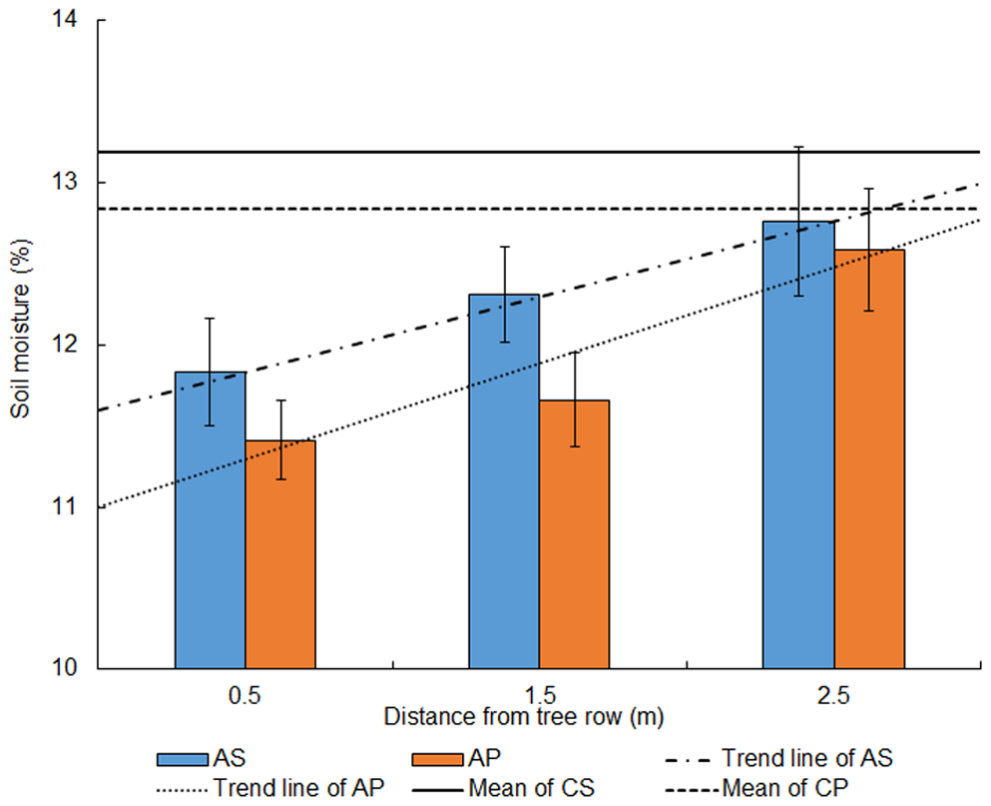
Soil moisture of 0 to 100 cm depth for the intercropping systems and its control. Vertical lines indicate confidence interval at 95% level.

Although the soil moisture content in the whole soil profile (0 to 100 cm in depth) in AS was different from AP, the trend of spatial distributions of soil moisture was similar (Figure 5). Soil moisture content in AS was related to distance from the apple tree row and showed a clear linear relationship (*Y* = 0.465×+11.602, *R* ^2^ = 0.999), and AP showed the same trend (*Y* = 0.590×+11.002, *R* ^2^ = 0.900). Compared with AP, AS had higher values at the same distance away from the tree row. However, with increasing distance from the tree row, soil moisture in AP had a higher growth than that in AS. The lowest soil moisture content was 11.83% in AS and 11.41% in AP, showed a decrease of 10.31% and 11.14% when compared with the corresponding control treatments. The soil moisture at F2.5 in both intercropping systems also had slightly lower values than that in monoculture configurations, however no difference was observed at significance level of 5%, since the control treatments mean fell within the confidence intervals of F2.5 in the corresponding intercropping systems (confidence level 95%). Otherwise, the average soil moisture content in AP was lower than that in AS.

### Spatial Distribution of Soil Nutrients

The soil nutrients content in the 0 to 100 cm interval was calculated (Table 2). It represented that organic matter, total N, available P and available K in AS had different degrees of reduction when compared with CS, and showed significant differences (*P*<0.05). Similar results were found between AP and CP, except that no significant difference was observed for total N and available K at the location of F2.5. The average content of organic matter, total N, available P and available K in AS decreased by 30.77%, 63.24%, 56.08% and 27.83% when compared with CS–the monoculture configuration. For AP and CP, the decreased percentages were 18.32%, 21.05%, 36.27% and 7.49% respectively. In addition, except available K, soil nutrients content in AP was higher than that in AS at the same spatial location. With the increasing distance from tree row, the distribution trend of soil nutrients was different from that of PAR, NPR or soil moisture in the same intercropping condition. The lowest content of organic matter, total N, available P and available K in AS were present at the location of F1.5. The similar result was found in AP, except that the lowest value of available K was the location of F0.5.

**Table 2:** Soil nutrients for the intercropping systems and control configurations.

Measurement	AS			CS	AP			CP
	F0.5	F1.5	F2.5		F0.5	F1.5	F2.5	
Organic matter (g•kg^−1^)	4.63±0.27a	3.40±0.29b	4.93±0.27a	6.24±0.31c	6.26±0.36a	5.21±0.26b	6.37±0.36a	7.28±0.27c
Total N (g•kg^−1^)	0.28±0.05a	0.22±0.04b	0.25±0.04ab	0.68±0.03c	0.31±0.06a	0.24±0.05b	0.35±0.07ac	0.38±0.04c
Available P (mg•kg^−1^)	2.82±0.56a	2.50±0.54a	3.93±0.31b	7.02±0.22c	5.06±0.61a	3.44±0.44b	4.75±0.42a	6.93±0.33c
Available K (mg•kg^−1^)	97.33±1.77a	84.02±2.09b	95.43±2.97a	127.84±2.75c	87.45±2.81a	90.73±2.45a	98.11±2.42b	99.55±2.19b

Data were given as the means ± SD.

Different lowercase letters within a row of each crop indicate significant differences (LSD, *P*<0.05).

### Crop Growth and Yields

Plant height, hundred leaf dry weight and total above-ground biomass in both intercropping systems had lower values when compared with the monoculture configuration (*P*<0.05; Table 3). The locations of F0.5 and F1.5 in all these parameters showed significant differences with corresponding monoculture configuration (*P*<0.05); however, there were no difference observed in the location of F2.5.

**Table 3:** Crop growth, biomass and yield for soybean and peanut intercropped with apple trees and its control.

Measurement	AS			CS	AP			CP
	F0.5	F1.5	F2.5		F0.5	F1.5	F2.5	
Crop height (cm)	43.7±2.8a	44.4±2.3a	47.7±3.2b	50.2±2.6b	19.1±0.7a	21.9±1.0b	22.6±0.9bc	23.5±0.8c
Hundred leaf dry weight (g)	14.12±0.81a	14.14±0.51a	14.95±0.38b	15.37±0.57b	4.39±0.23a	5.79±0.22b	5.90±0.31bc	6.14±0.28c
Total above-ground biomass (g)	54.55±4.66a	66.06±3.81b	76.96±4.30c	79.76±4.22c	50.10±2.62a	51.57±2.45a	79.16±3.28b	79.65±2.46b
Yield (t/ha)	1.48±0.06a	1.69±0.04b	1.84±0.06c	1.91±0.04c	1.62±0.04a	1.66±0.04a	1.81±0.05b	1.86±0.04b

Data were given as the means ± SD.

Different lowercase letters within a row of each crop indicate significant differences (LSD, *P*<0.05).

The yield of soybean in AS was significantly related to the distance from the row of apple trees (*Y* = 0.180×+1.400, *R* ^2^ = 0.991), and the yield of peanut in AP showed the same trend (*Y* = 0.095×+1.554, *R* ^2^ = 0.900) which showed that yield of soybean had greater impacted by distance from the tree row. The yields at F0.5 and F1.5 in AS were lower than that in CS (*P*<0.05), with a reduction of 22.45% and 11.95%, and in AP the yields of reduction were 13.31% and 11.03% when compared with CP. No differences were observed between the locations of F2.5 in both intercropping systems and the corresponding monoculture configuration (*P*<0.05).

Within plot differences in these parameters were significantly correlated (Table 4). NPR was highly correlated with PAR, and soil moisture. The total above-ground biomass of soybean was highly correlated with PAR, soil moisture and available P. The total above-ground biomass of peanut was correlated with PAR, soil moisture, total N and available P. Yield of soybean was highly correlated with PAR, soil moisture, available P and total N, with a trend of soil moisture >PAR>available P>total N. For peanut, the trend was soil moisture > PAR > total N > available P. It showed that, for both of the intercropping system in the study region, the primary factor affecting the yield is soil moisture, and the secondary factor is photosynthetically active radiation, and soil nutrient also have an impact on crop yield in some depth.

**Table 4:** Correlations of soybean and peanut net photosynthetic rate, biomass, and yield with environmental or physiological parameters measured in Jixian, China.

Independent variable	NPR ( µmol·s^−1^·m^−2^)	Total above-ground biomass (g)	Yield (kg/ha)
Soybean			
PAR ( µmol·s^−1^·m^−2^)	0.973**	0.996**	0.952**
Soil moisture (%)	0.953**	0.977**	0.957**
Organic matter (g•kg^−1^)	0.441	0.575	0.566
Total N (g•kg^−1^)	0.537	0.555	0.601*
Available P (mg•kg^−1^)	0.697*	0.750**	0.763**
Available K (mg•kg^−1^)	0.469	0.538	0.565
Peanut			
PAR ( µmol·s^−1^·m^−2^)	0.986**	0.773**	0.816**
Soil moisture (%)	0.926**	0.965**	0.959**
Organic matter (g•kg^−1^)	0.450	0.562	0.583
Total N (g•kg^−1^)	0.513	0.843**	0.770**
Available P (mg•kg^−1^)	0.424	0.628*	0.646*
Available K (mg•kg^−1^)	0.479	0.531	0.446

* Significant at 5% level.

** Significant at 1% level.

## Discussion

Agroforestry system has been studied for a long time and has been widely used in the agricultural production practices in China [3], [16], [17]. However, there has been little research done on the agroforestry system in the Loess Plateau region. The main intercropping models which have been studied are always walnut-wheat and apple-wheat [18]–[20]. The types of fruit trees intercropping with economic crops such as soybean and peanut has not been well studied. In fact, compared with wheat, soybean and peanut could bring more economic income to farmers. At the same time, these two crops could be rotated with wheat in order to improving land-use efficiency, and re-establishing the economic viability of the Loess Plateau.

Our study observed a clearly positive linear relationship between distance from the apple tree rows and the daily mean values of PAR and NPR in the intercropping systems. For both apple-crop intercropping systems, the shading of the 4–5 years old apple trees had a significant negative effect on the crops in the range of 1.5 m away from the tree rows and further caused the reduction of crop yield. In other researches of temperate agroforestry systems, the similar results were reported by Reynolds et al. [21] about maize and soybean intercropped with poplar and silver maple in Canada and Peng et al. [22] about mungbean and pepper intercropped with walnut and plum in Weibei area, China. For total above-ground biomass and yield of both crops, PAR of soybean had higher correlations than that of peanut, which indicated that soybean is more adversely impacted by tree shading. Within tree-based intercropping systems, many factors such as tree species, tree height, crown shape, tree row orientation and distance between tree rows can influence tree shading of adjoining agricultural crops. Light reduction would depend on the extent and duration of the shade of trees [21]. Regular pruning of fruit trees could reduce light competition within the intercropping system, improving crop yields.

In semiarid and arid regions, it is still a focus of studies whether intercropping system has an overall negative or positive effect on soil moisture [23]. In some related studies, it was considered that the trees can improve soil moisture holistic conditions in intercropping systems [17], [24]. In other studies, the opposite results were reported [11], [12], [25], [26]. However, little research has been carried out in this aspect on the Loess Plateau. Our research confirmed that the competition of water between trees and crops do exist, and showed adverse effects in the study site. A clear linear relationship was observed between the distance from the tree row and soil moisture in both of the intercropping systems. The closer to the tree row, the more intense the competition. The lowest soil moisture content in apple-soybean intercropping system and apple-peanut intercropping system showed a reduction of 10.31% and 11.14%, respectively. Only considering competition of water, the mainly affected region of the apple trees was 1.5 m away from the tree rows under the current tree age.

Another key factor of crop growth is soil nutrients in the intercropping systems. Elsewhere, Thomas et al. [27] and Thevathasan et al. [13] have reported that competition for nutrients in intercropping systems does not exist. In our study, it identified that there were competition for soil nutrients between trees and crops in the intercropping systems. The average content of organic matter, total N, available P and available K showed different degrees of reduction in both of the apple-crops intercropping systems than that of the corresponding control treatments. In particular, total N and available P had higher reduction rate than organic matter and available K, and had significantly correlation with yield of crops. As leguminous plants, soybean and peanut could fix nitrogen from the air via a symbiotic relationship with rhizobium bacteria and increase the mineral soil nitrogen content [28], [29]. However, the nitrogen coming from biologically fixed N_2_ of symbiosis could not meet all the demand of crops growth, and any gaps between N supply by N_2_ fixation and crop N demand must be met by N uptake from soil [30]. The deficiency of light and water in the intercropping systems reduced the physiological activity of the crop, and then affected the N fixation capacity, resulting more intense competition for nitrogen between trees and crops. Compared with soybean and peanut, the growth of other non-nitrogen-fixing crop species (i.e. wheat, maize and millet) would be more severely affected because of nitrogen deficiency in the intercropping systems. Different from understory light distribution and soil moisture, soil nutrients had a different variation pattern in both intercropping systems. The main reasons for this phenomenon might be: (1) the crops close to tree row were seriously affected by tree shading, soil moisture stress and human activities, resulting in low physiological activity and low absorption of soil nutrients; (2) the decomposition of tree litter leaded to high nutrients content in the area near the tree row; (3) the overlapping of apple tree roots and crop roots resulting in lower nutrient content at F1.5; (4) the tree roots reduced with the increase of the distance from the tree, therefore, the soil nutrients had relatively high content F2.5. Therefore, in the area of 1.5 m away from tree row, strengthen the application of fertilizer (especially nitrogen and phosphorus) would be helpful to alleviate interspecific competition for soil nutrients.

In the apple-crop intercropping systems, the competition of light, water and nutrients resulted in a greater negative impact on crop growth and yields. For the two apple-crop intercropping systems in our study, the primary factor affecting the crop yield was soil moisture, and the secondary factor was light, and deficiency of the soil nutrient also had a negative impact on crop yields. In the same study area, Yun et al. reported a similar research with a different conclusion: the light is the primary limiting factor leading to reduction of crops, followed by soil moisture [31]. In their research, the apple trees had greater crown width, canopy density and root depth due to elder age (9-year-old) and smaller tree spacing (3 m×4 m). Affected by the impact of canopy structure, the obvious microclimate effect inhibited evapotranspiration of soil moisture to some extent [32], in the same time, the low transmittance led to more intense light stress to crops. Furthermore, the effect of hydraulic lift by tree roots also alleviated the interspecific competition for soil water [24]. Combined with these reasons, different results were found. For different intercropping patterns and tree ages, the intensity of competition for resources would be different in the intercropping system. Therefore, a long-term observation should be carried out in this region to obtain more details about the mechanism of interspecific competition in the intercropping systems. In our study, under the current tree age and growth conditions, the influence scope of the apple trees was 1.5 m away from the tree rows. Compared with the corresponding monoculture configuration, the yield of peanut in the intercropping system had a lower reduction than that of soybean. With comprehensive consideration, peanut is more suitable for intercropping with apple trees in this region.

As we have demonstrated in this study, soil moisture, light and soil nutrients were the limiting factors of crop yield. In order to obtain more production, appropriate management measures were needed to minimize competition between trees and crops. Namirembe [33] and Friday [6] have suggested that the competition for light between trees and crops could be alleviated by pruning of trees crown and increasing the intercropping distance. In general, the aboveground competition could be intuitively observed and managed. However, the competition belowground is invisible and easily ignored by farmers or managers. To avoid these yield losses, root barrier in the intercropping interface is considered to be a useful agricultural management practice according to some related studies [12], [34], [35]. Combined with their research achievements and our experiment result, we offered several specific recommendations to reduce the competition exist in apple-crop intercropping systems: (1) the selection of crop varieties which is more suitable for apple-crop intercropping systems; (2) appropriate distance increase between the crops and apple tree rows; (3) regular pruning of fruit trees, in order to increase canopy light transmittance rate; (4) additional fertilization and irrigation in the key phenological phase of the crops; (5) differences of irrigation and fertilization based on the distance from the apple trees. Management measures such as plastic film and straw mulching have been widely used in agricultural production. Whether these measures have overall positive effects on intercropping system would be one of the focus of our future research work in this region.

## Conclusions

As an effective method to increase the efficiency of land use and economic returns, tree-based intercropping systems are particularly important on Loess Plateau. We concluded that the competitions exist both above-ground and below-ground between apple trees and crops. The competition for soil moisture is the primary limiting factor for the crop productivity in this region. Furthermore, the tree shading and the competition for soil nutrients in the interface of trees and crops also have a negative impact on the understory crops. However, it could be minimized by better agricultural technology and management measures.

In summary, our study suggests that there is great potential for intercropping systems in the Loess Plateau. Therefore, in order to relieve the shortage of arable land and promote the sustainable development of natural resources, the intercropping systems would continue to be the hot spot for future research. Canopy structure, roots distribution of trees, the application of different agronomic measures and the role they play in the competition process in the intercropping systems will be the focus of our future research.

## Supporting Information

Figure S1 **Picture of apple-soybean intercropping plots in study site.**
(JPG)

Figure S2 **Picture of peanut monoculture plots in study site.**
(JPG)

## References

[1] Burel F (1996) Hedgerows and their role in agricultural landscapes. Critical Reviews in Plant Sciences 15: 169–190.

[2] Gene Garrett HE, Buck L (1997) Agroforestry practice and policy in the United States of America. Forest Ecology and Management 91: 5–15.

[3] Li W, Lai S (1994) Agroforestry in China. Beijing: Chinese Science Press. pp. 14–18.

[4] Zhu Q, Zhu J (2003) Sustainable management technology for conversion of cropland to forest in loess area. Beijing:Chinese Forestry Press. pp. 160–165.

[5] Ong CK, Huxley P (1996) Tree-crop interactions: a physiological approach. Wallingford:CAB International. pp. 386.

[6] Friday JB, Fownes JH (2002) Competition for light between hedgerows and maize in an alley cropping system in Hawaii, USA. Agroforestry Systems 55: 125–137.

[7] Peng X, Zhang Y, Cai J, Jiang Z, Zhang S (2009) Photosynthesis, growth and yield of soybean and maize in a tree-based agroforestry intercropping system on the Loess Plateau. Agroforestry Systems 76: 569–577.

[8] Hall DJM, Sudmeyer RA, McLernon CK, Short RJ (2002) Characterisation of a windbreak system on the south coast of Western Australia. 3. Soil water and hydrology. Australian Journal of Experimental Agriculture 42: 729–738.

[9] Unkovich M, Blott K, Knight A, Mock I, Rab A, et al (2003) Water use, competition, and crop production in low rainfall, alley farming systems of south-eastern Australia. Australian Journal of Agricultural Research 54: 751–762.

[10] Kowalchuk TE, Jong E (1995) Shelterbelts and their effect on crop yield. Canadian Journal of Soil Science 75: 543–550.

[11] Jose S, Gillespie AR, Seifert JR, Biehle DJ (2000) Defining competition vectors in a temperate alley cropping system in the midwestern USA: 2. Competition for water. Agroforestry Systems 48: 41–59.

[12] Miller AW, Pallardy SG (2001) Resource competition across the crop-tree interface in a maize-silver maple temperate alley cropping stand in Missouri. Agroforestry Systems 53: 247–259.

[13] Thevathasan NV, Gordon AM, Simpson JA, Reynolds PE, Price G, et al (2004) Biophysical and ecological interactions in a temperate tree-based intercropping system. Journal of Crop Improvement 12: 339–363.

[14] Newman SM, Bennett K, Wu Y (1997) Performance of maize, beans and ginger as intercrops in Paulownia plantations in China. Agroforestry Systems 39: 23–30.

[15] Yun L, Bi H, Gao L, Zhu Q, Ma W, et al (2012) Soil moisture and soil nutrient content in walnut-crop intercropping systems in the Loess Plateau of China. Arid Land Research and Management 26: 285–296.

[16] Meng P, Zhang J, Fan W (2003) Research on agroforestry in china. Beijing:Chinese Forestry Press. pp.235.

[17] Meng P, Zhang J (2004) Effects of pear-wheat inter-cropping on water and land utilization efficiency. Forest Research 17: 167–171.

[18] Zhang J, Meng P, Yin C (2002) Spatial distribution characteristics of apple tree roots in the apple-wheat intercropping. Scientia Silvae Sinicae 38: 30–33.

[19] Zhang J, Meng P (2004) Model on wheat potential evapotranspiration in apple-wheat intercropping. Forest Research 17: 284–290.

[20] Yun L, Bi H, Ren Y, Ma W, Tian X (2009) Soil moisture distribution at fruit-crop intercropping boundary in the Loess region of Western Shanxi. Journal of Northeast Forestry University 37: 70–78.

[21] Reynolds PE, Simpson JA, Thevathasan NV, Gordon AM (2007) Effects of tree competition on corn and soybean photosynthesis, growth, and yield in a temperate tree-based agroforestry intercropping system in southern Ontario, Canada. Ecological Engineering 29: 362–371.

[22] Peng X, Cai J, Jiang Z, Zhang Y, Zhang S (2008) Light competition and productivity of agroforestry system in loess area of Weibei in Shaanxi. Chinese Journal of Applied Ecology 19: 2414–2419. 19238840

[23] Zhang J, Meng P, Yin C, Cui G (2003) Summary on the water ecological characteristics of agroforestry system. World Forestry Research 16: 10–14.

[24] Hirota I, Sakuratani T, Sato T, Higuchi H, Nawata E (2004) A split-root apparatus for examining the effects of hydraulic lift by trees on the water status of neighbouring crops. Agroforestry Systems 60: 181–187.

[25] Lott JE, Howard SB, Ong CK, Black CR (2000) Long-term productivity of a Grevillea robusta-based overstorey agroforestry system in semi-arid Kenya: II. Crop growth and system performance. Forest Ecology and Management 139: 187–201.

[26] Lehmann J, Peter I, Steglich C, Gebauer G, Huwe B, et al (1998) Below-ground interactions in dryland agroforestry. Forest Ecology and Management 111: 157–169.

[27] Thomas J, Kumar BM, Wahid PA, Kamalam NV, Fisher RF (1998) Root competition for phosphorus between ginger and Ailanthus triphysa in Kerala, India. Agroforestry Systems 41: 293–305.

[28] Cheng D (1994) Resource microbiology. Harbin:Northeast Forestry University Press. pp. 32.

[29] Wani SP, Rupela OP, Lee KK (1995) Sustainable agriculture in the semi-arid tropics through biological nitrogen fixation in grain legumes. Plant Soil 174: 29–49.

[30] Salvagiotti F, Cassman KG, Specht J E, Walters DT, Weiss A, et al (2008) Nitrogen uptake, fixation and response to fertilizer N in soybeans: A review. Field Crops Research 108: 1–13.

[31] Yun L, Bi H, Tian X, Cui Z, Zhou H, et al (2011) Main interspecific competition and land productivity of fruit-crop intercropping in Loess Region of West Shanxi. Chinese Journal of Applied Ecology 22: 1225–1232. 21812299

[32] Zhang J, Meng P, Song Z, Gao J (2004) An overview on micro-climatic effects of agro-forestry systems in plain agricultural areas in China. Agricultural Meteorology 25: 52–55.

[33] Namirembe S (1999) Tree management and resource utilization in agroforestry systems with Senna spectabilis in the drylands of Kenya. Bangor:University of Wales. pp. 206.

[34] Singh RP, Saharan N, Ong CK (1989) Above and below ground interactions in alley-cropping in semi-arid India. Agroforestry Systems 9: 259–274.

[35] Hou Q, Brandle J, Hubbard K, Schoeneberger M, Nieto C, et al (2003) Alteration of soil water content consequent to root-pruning at a windbreak/crop interface in Nebraska, USA. Agroforestry Systems 57: 137–147.

