# Supplementary figures and images for: Intercropping Competition between Apple Trees and Crops in Agroforestry Systems on the Loess Plateau of China
Source: PLoS One. 2013 Jul 25;8(7):e70739. doi: 10.1371/journal.pone.0070739 (PMC3723670; doi:10.1371/journal.pone.0070739)

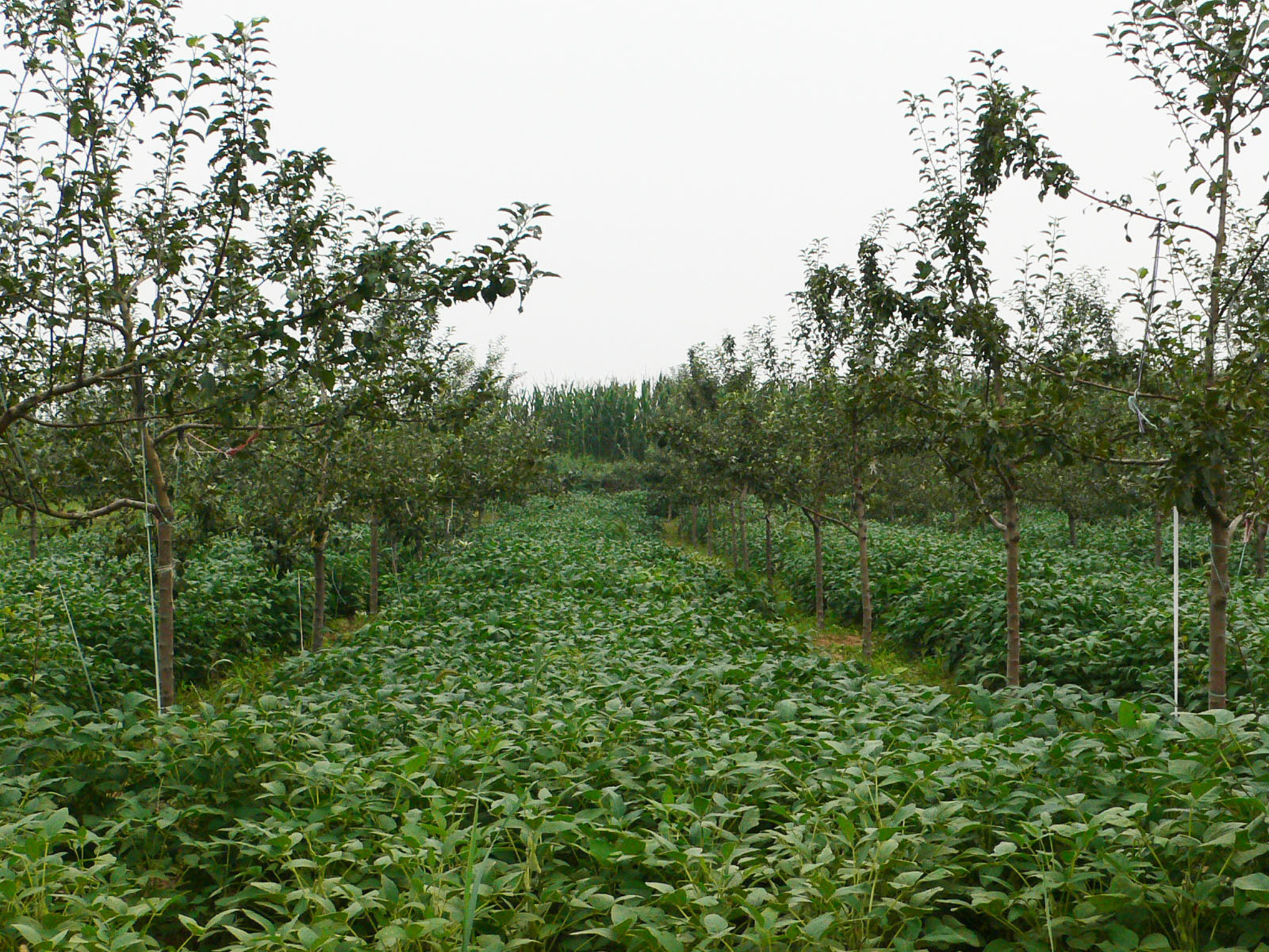

Supplement: Figure S1 — Picture of apple-soybean intercropping plots in study site. (JPG) [file pone.0070739.s001.jpg]

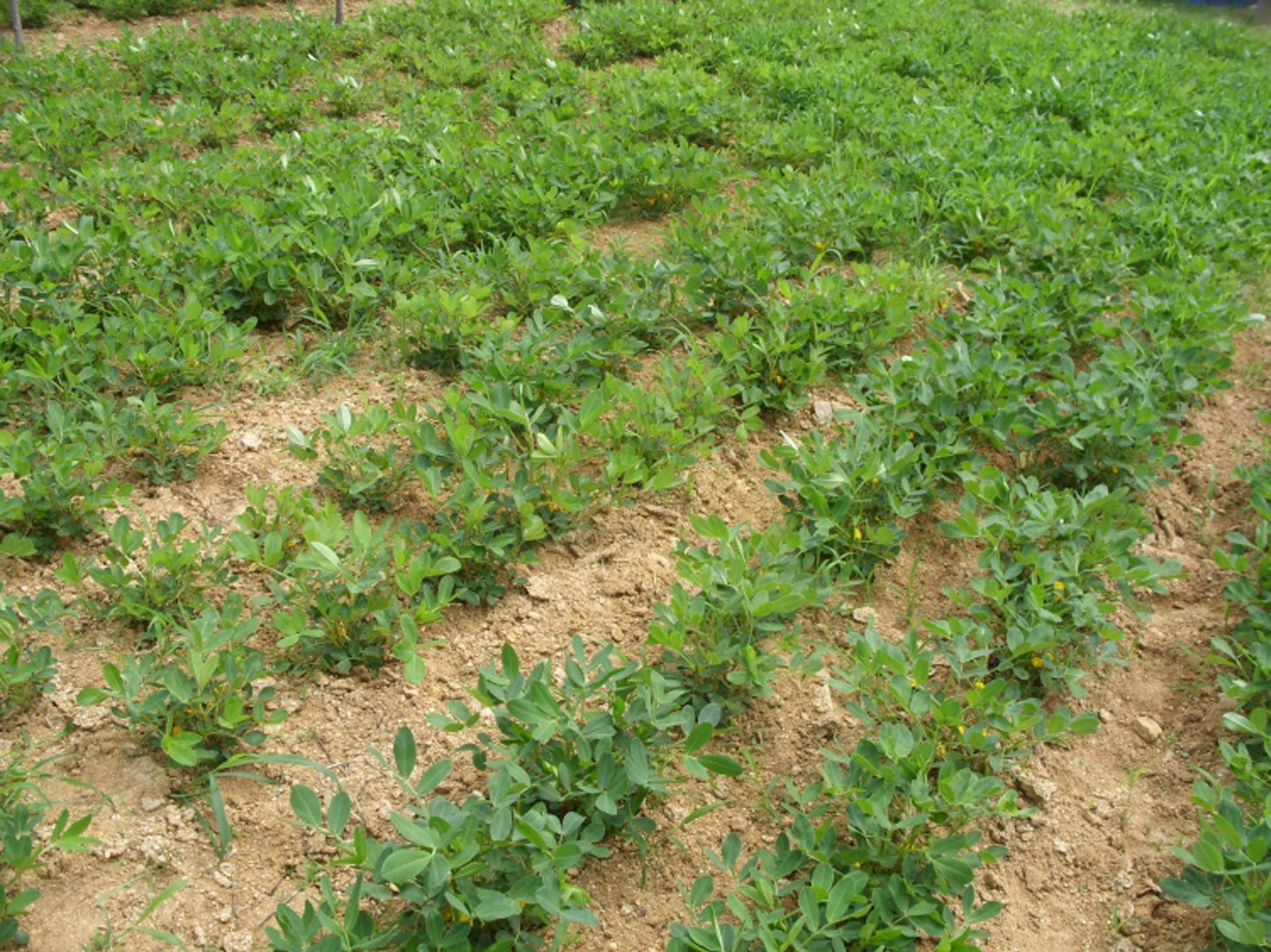

Supplement: Figure S2 — Picture of peanut monoculture plots in study site. (JPG) [file pone.0070739.s002.jpg]
